# Prenatal Use of Exome Sequencing and Chromosomal Microarray Analysis: Indications, Interpretation, and Gene Selection Strategies

**DOI:** 10.3390/diagnostics16020185

**Published:** 2026-01-07

**Authors:** Laia Rodriguez-Revenga, Victoria Ardiles-Ruesjas, Antoni Borrell

**Affiliations:** 1 Biochemistry and Molecular Genetics Department (CDB), Hospital Clínic of Barcelona, 08036 Barcelona, Spain; 2 Fundació de Recerca Clínic Barcelona-Institut d’Investigacions Biomediques August Pi i Sunyer (FRCB-IDIBAPS), 08036 Barcelona, Spain; 3 CIBER of Rare Diseases (CIBERER), Instituto de Salud Carlos III, 28029 Madrid, Spain; 4 Fetal Medicine Research Center, BCNatal—Barcelona Center for Maternal-Fetal and Neonatal Medicine, Institut Clínic de Ginecologia, Obstetricia i Neonatologia, Hospital Clínic de Barcelona, 08028 Barcelona, Spain; 5 Department of Medical-Surgical Specialties, Medicine and Health Sciences School, Universitat de Barcelona, 08036 Barcelona, Spain

**Keywords:** prenatal diagnosis, exome sequencing, chromosomal microarray analysis, clinical relevance, diagnosis, genetic abnormalities

## Abstract

As genomic technologies continue to evolve, understanding the scope and limitations of available prenatal testing methods is essential for accurate diagnosis and counseling. Chromosomal microarray analysis (CMA) and exome sequencing (ES) have emerged as key complementary tools in this setting. This review aims to outline the technical principles underlying CMA and ES and to compare their diagnostic capabilities and limitations in the prenatal context. This narrative review includes a literature search, with additional relevant articles identified through manual screening of reference lists from key publications and review articles. Due to the narrative nature of this review, no formal inclusion or exclusion criteria or quantitative synthesis were applied. Special focus was placed on clinical indications, variant interpretation challenges—particularly uncertain and incidental findings—gene selection strategies, and implications for prenatal counseling. Indications for both tests have increased over time but differ substantially. CMA is becoming the standard prenatal genetic test, particularly in the evaluation of fetal structural anomalies, whereas ES remains restricted to selected fetal structural anomalies. Interpretation of molecular results remains a major challenge, especially for variants of uncertain significance and incidental findings with unclear or unexpected implications for pregnancy management. For ES, agnostic gene selection strategies showed superior diagnostic yield compared with phenotype-driven approaches, likely reflecting the limited characterization of prenatal phenotypes. Continuous refinement of clinical indications, bioinformatic pipelines, variant classification criteria, and gene curation strategies is critical to ensure that prenatal results are accurate and clinically meaningful. Together, ongoing improvements in technology, interpretation, and clinical integration have the potential to transform prenatal genomics into a more precise, informed, and ethically responsible field.

## 1. Introduction

Prenatal diagnosis has evolved considerably over time, advancing from basic imaging and cytogenetic analyses to sophisticated molecular and genomic techniques that enable earlier and more precise detection of fetal abnormalities. The advent of high-throughput methods such as chromosomal microarray analysis (CMA) and next-generation sequencing (NGS) represents a major milestone in this evolution, allowing for simultaneous analysis of numerous genes and detection of diverse pathogenic variants in a cost-effective manner. NGS can be applied to targeted gene panels, exome sequencing (ES), or whole-genome sequencing (WGS). While traditional methods, including karyotyping, quantitative fluorescence-PCR (QF-PCR), and CMA, can detect approximately 40% of genetically driven fetal anomalies, NGS has substantially increased the diagnostic yield, identifying pathogenic variants in 10–40% of cases depending on the type of malformation or organ system evaluated [1].

CMA and ES have emerged as complementary technologies, each with distinct strengths and limitations. Understanding the technical principles, diagnostic capabilities, and clinical applications of these methods is essential for accurate prenatal diagnosis, informed counseling, and optimal patient management. Beyond technical performance, accurate clinical interpretation, standardized variant classification, and thoughtful reporting strategies are critical to ensure that molecular findings are meaningful and actionable in the prenatal setting.

This narrative review explores both the technical and clinical aspects of prenatal CMA and ES, highlighting their indications, interpreting challenges and gene selection strategies.

## 2. Methods

A systematic review methodology was not employed. A literature search was conducted using PubMed, applying combinations of keywords including “prenatal diagnosis,” “chromosomal microarray,” “exome sequencing,” “prenatal exome,” and “fetal anomalies.” Additional relevant articles were identified through manual screening of reference lists from key publications and review articles. Peer-reviewed articles published primarily in English were considered, with an emphasis on studies, guidelines, and reviews relevant to prenatal clinical practice and molecular diagnostics. Due to the narrative nature of this review, no formal inclusion or exclusion criteria or quantitative synthesis were applied. As this review is based entirely on previously published data, no new data were collected and ethical approval was not required. All data were used solely for the purposes of this review. Special focus was placed on clinical indications, variant interpretation challenges—particularly uncertain and incidental findings—gene selection strategies, and implications for prenatal counseling.

## 3. Technical Principles

### 3.1. Chromosomal Microarray Analysis

CMA is a genomic technique used to detect DNA copy number variations (CNVs), which are a form of structural genomic variation characterized by the gain or loss of DNA. CNVs are currently recognized as the greatest source of genomic diversity in humans and its presence does not inherently imply pathogenicity. The clinical significance of a CNV depends largely on its size, genomic location, and the functional relevance of the genes involved. Benign CNVs are typically small, occur in regions of the genome with low gene density, or involve genes with limited phenotypic impact. In contrast, pathogenic CNVs often disrupt dosage-sensitive genes, regulatory elements, or critical genomic regions, leading to altered gene expression and disease phenotypes.

In prenatal diagnosis, CMA is widely established as a first-tier test for fetuses presenting with structural anomalies as it offers higher resolution than traditional karyotyping and can identify submicroscopic abnormalities that are not visible under a microscope [1,2]. While standard karyotyping typically detects alterations exceeding 7–10 Mb in size, CMA enables the identification of imbalances (microdeletions or microduplications) at a much higher resolution, often within the kilobase range [3].

There are two main types of CMA platforms: array comparative genomic hybridization (aCGH) and single nucleotide polymorphism (SNP) arrays. aCGH works by comparing fetal DNA to a reference sample to detect differences in DNA copy number, using fluorescently labeled probes across the genome. It is highly effective in identifying deletions and duplications but cannot detect copy-neutral abnormalities such as uniparental disomy (UPD). SNP arrays, on the other hand, use probes targeting specific single nucleotide polymorphisms to detect CNVs and provide genotype information. This allows SNP arrays to identify copy-neutral events like UPD where both copies of a chromosome come from one parent, and regions of homozygosity, which may indicate consanguinity or maternal cell contamination. While both types of arrays are valuable, SNP arrays offer additional insights into genomic structure beyond CNVs [4].

### 3.2. Exome Sequencing

Prenatal exome sequencing (ES), on the other hand, targets the protein-coding regions (exons) of the genome which are most likely to contain disease-causing mutations. ES enables the detection of single nucleotide variants (SNVs) and small insertions or deletions (indels), making it particularly valuable in cases where CMA yields normal results, but a monogenic disorder is suspected. These alterations are often responsible for fetal structural anomalies when chromosomal tests like karyotyping or microarray yield normal results [5,6]. A comparison between CMA and ES in the context of prenatal diagnosis is shown in Table 1.

ES begins with the extraction of high-quality genomic DNA from a biological sample such as uncultured amniotic fluid. The DNA is then fragmented and equipped with sequencing adapters to create a library. Using hybridization-based capture, fragments corresponding to protein-coding regions (exons) are selectively enriched. These regions comprise about 1–2% of the human genome but contain the majority of known disease-causing variants. The enriched libraries are sequenced on a high-throughput platform, generating millions of short reads. These reads are aligned to a reference genome, and computational pipelines are applied to identify genetic variants. Finally, the detected variants are annotated using functional, population frequency, and clinical databases to support their interpretation and reporting in clinical context.

Prenatal ES has become increasingly available in many countries and current guidelines support its use in certain high-risk circumstances, specifically in those pregnancies complicated by fetal anomalies not explained by karyotype or CMA [7]. In prenatal settings, ES is especially useful for detecting pathogenic or likely pathogenic variants in genes associated with the fetal phenotype. Although the diagnostic yield of ES varies significantly by indication, meta-analyses studies describe a molecular diagnostic rate of about 20–30% [8,9]. ES is not only capable of diagnosing suspected genetic disorders but also has the potential to uncover a wide range of diseases that historically went undiagnosed in utero. This capacity to detect rare, novel, or unexpected variants has transformed the diagnostic landscape, enabling earlier interventions, improved clinical management, and more informed decision-making for families.

However, ES has limitations. It does not detect large deletions or duplications (unless combined with CNV-calling algorithms), or structural rearrangements, nor does it identify mitochondrial DNA variants, repeat expansions, or epigenetic changes. Furthermore, it can also report variants of uncertain significance (VUS) particularly when alterations are observed in genes relevant to the clinical phenotype, thereby complicating interpretation.

In summary, CMA is best for detecting large-scale chromosomal changes, while ES excels at identifying single-gene disorders. They are complementary tools, and in complex cases, both may be used to increase diagnostic yield. In a near future, as sequencing costs continue to decline and bioinformatics methods become more sophisticated, genome sequencing (GS) is expected to surpass both CMA and ES in diagnostic yield. GS offers a unified and comprehensive approach, capable of detecting a wide range of genetic variants (including CNVs and SNVs) within a single test.

## 4. Clinical Indications

### 4.1. Indications of CMA

CMA has become the standard prenatal diagnostic test in most centers, largely replacing conventional karyotyping regardless of the indication for sampling. Exceptions remain, however, including cases referred due to known familial chromosomal rearrangements and situations in which trisomy 21 or trisomy 13 is detected by QF-PCR or FISH, where karyotyping is required to confirm or exclude the presence of Robertsonian translocations. Even in late amniocentesis, when fetal infection or cystic fibrosis is suspected due to echogenic bowel, CMA is increasingly offered to pregnant women and couples.

If CMA is not the routine test in prenatal diagnosis, it should be the elective test in the following cases:

1. Fetal structural abnormality on ultrasound: detection of a major malformation, more than one minor malformation, or markers/findings suggestive of birth defects.
2. Fetal growth restriction (FGR): defined as estimated fetal weight/biometrics < 3rd percentile in isolated presentation before 24 weeks, or associated with any ultrasound marker (including femur length < −3 SD and polyhydramnios, but not oligohydramnios) before 28 weeks.
3. Increased nuchal translucency (>99th percentile) at 11–13 weeks.
4. Previous child with a cryptic ‘de novo’ deletion or duplication. Given the possibility of germline mosaicism in one parent, targeted FISH or MLPA may also be used, but CMA is recommended due to its genome-wide coverage in invasive testing.
5. Family history of chromosomal rearrangement at risk in the ongoing pregnancy:
  a Balanced reciprocal parental translocation or pericentric inversion.
  b Familial cryptic deletion or duplication with significant transmission risk, penetrance, and clinical relevance.
  c Marker chromosome mosaicism in one parent, potentially pathogenic if inherited by the fetus.
6. Characterization of specific findings in the fetal karyotype:
  a Reciprocal translocation.
  b Apparently balanced ‘de novo’ inversion.
  c Marker chromosome (particularly ring-type and non-satellite markers).
7. Second-trimester pregnancy loss or intrauterine fetal demise: as cell culture is not required, CMA can be applied to non-viable cells that would not grow in culture, making it the technique of choice in these cases.

### 4.2. Indications of ES

**Figure 1:**
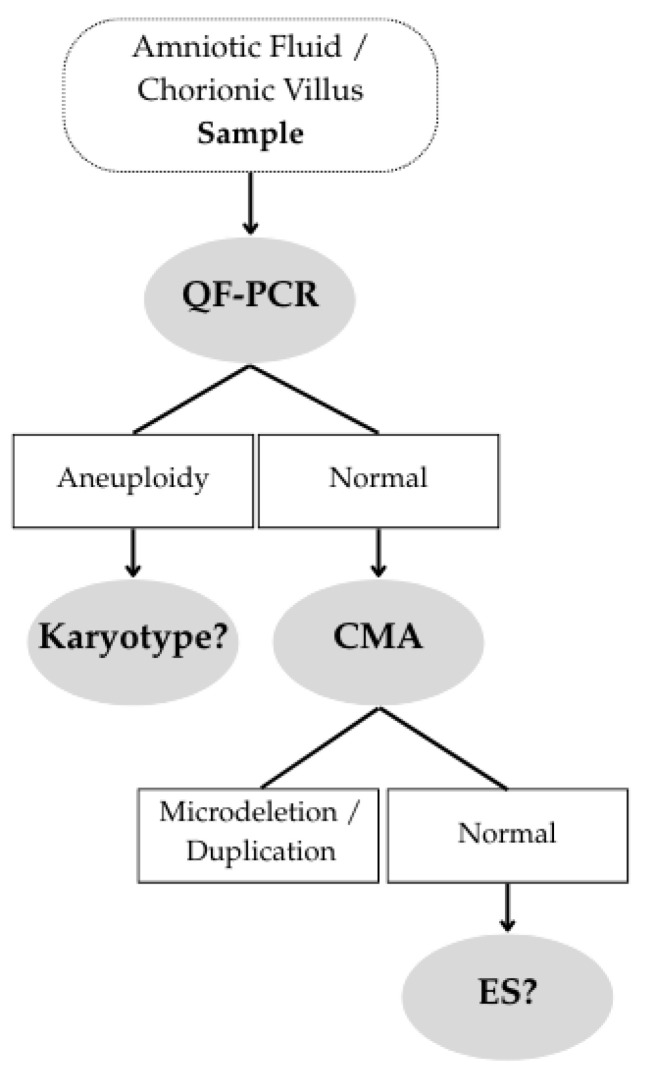
Clinical Decision Pathway.

Exome sequencing (ES) is an NGS-based technique that analyzes the coding exons of all genes, representing 1–2% of the genome but containing ~85% of known pathogenic variants. ES is used when a disease may be caused by variants across multiple possible genes. It is typically performed after a normal CMA result (see Figure 1), almost exclusively in selected cases of structural fetal anomalies.

In many centers, a multidisciplinary committee—ideally including a molecular geneticist, one or two clinical geneticists (prenatal and pediatric), a perinatal pathologist, and a genetic counselor—decides whether the phenotypic pattern of a specific case suggests a monogenic disorder.

Initially, multisystem anomalies (multiple anomalies involving different organ systems) were thought to provide the highest diagnostic yield. A meta-analysis by our group showed a 33% diagnostic yield across 17 studies, with Kabuki, CHARGE, and Smith-Lemli-Opitz syndromes being the most common [10,11]. Recurrent anomalies (similar anomalies across different pregnancies) were also expected to yield high results. Our meta-analysis showed a 40% yield across 9 series, with 80% having recessive inheritance, and 40% of those being homozygous. Meckel syndrome was the most frequent [10,11].

**Figure 2:**
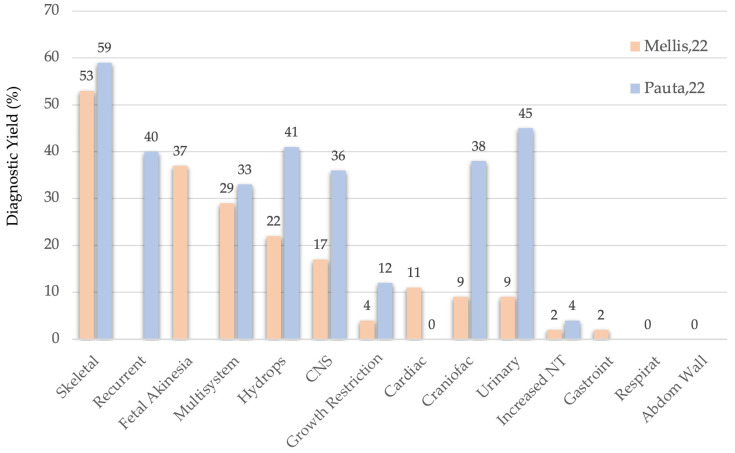
ES Diagnostic Yield according to Mellis’ and Pauta’s studies.

The anomaly type with the highest diagnostic yield has proven to be skeletal dysplasia, with a 55% yield according to Mellis’ large meta-analysis [8] and our own data (see Figure 2). In descending order of yield, the following anomaly types were observed: recurrent anomalies (40%), fetal akinesia/arthrogryposis (37%), multisystem anomalies (33%), and hydrops (41%). These five indications are widely accepted across groups [12]. Additionally, complex CNS anomalies (36%), craniosynostosis (38%), and echogenic large kidneys (45%) are also considered high-yield indications in our center.

If the diagnostic yield threshold were lowered from 20% to 10%, additional anomaly types would qualify: isolated CNS anomalies (17%), severe early-onset fetal growth restriction (12%), and cardiac defects (11%).

ISPD Guidelines [7]: Main Indications for ES.

1. A fetus with a major single anomaly or multiple anomalies where no genetic diagnosis was found after CMA, and expert clinical review suggests a possible monogenic etiology.
2. A history of a prior fetus (or child) with undiagnosed major anomalies, with recurrence of similar anomalies in the current pregnancy, and no genetic diagnosis after karyotype or CMA.
3. No evidence supports routine ES for other indications beyond fetal anomalies. Exceptions may include strong family history of severe, recurrent childhood-onset genetic conditions with no prenatal phenotype.

According to Rellys’ s review [12] the indications with a higher agreement between the 5 countries/regions that have set up a pre-established list of indications were the following:

1. Multisystem anomalies;
2. Non-immune hydrops fetalis;
3. Skeletal dysplasia;
4. Large bilateral echogenic kidneys;
5. Major CNS anomaly (excluding neural tube defects);
6. Multiple contractures/arthrogryposis;
7. Recurrent anomaly.

### 4.3. A Concurrent CMA-ES Approach

**Figure 3:**
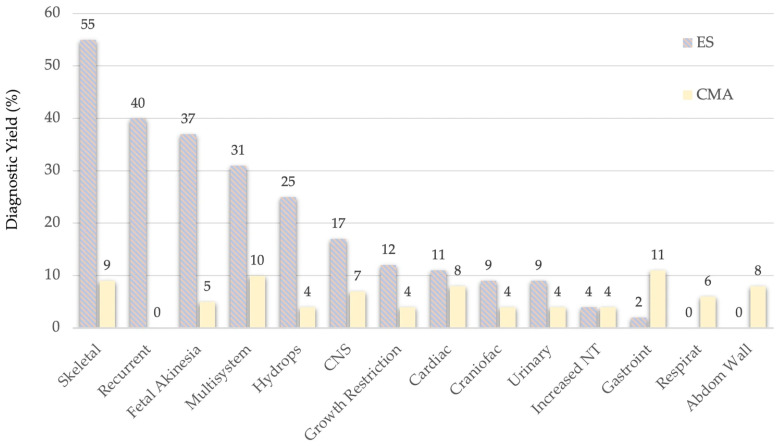
Comparison of ES and CMA’s Diagnostic Yield.

Interestingly, the mean calculated between Mellis and Pauta’s [10,11] diagnostic yield for ES (see Figure 3) and CMA [13], the diagnostic yield of the latter remains relatively stable across anomaly types (generally 4–9%, ranging 0–11%) [13].

If those anomaly types with a delta value higher that 20 percentual points between the CMA and ES diagnostic yields were selected, then 5 elective anomaly types for performing CMA concurrently with ES will be:

1. Skeletal dysplasia—46%
2. Recurrent anomalies—40%
3. Fetal akinesia—32%
4. Multisystem anomalies—21%
5. Hydrops—21%

## 5. Challenges in Variant Interpretation

To classify a single nucleotide variant (SNV), laboratory geneticists follow a structured workflow based on the American College of Medical Genetics and Genomics guidelines [14]. The process begins with variant detection through sequencing technologies, followed by initial annotation using bioinformatics tools to assess gene location, predicted impact, and population frequency. The variant is then compared against databases such as ClinVar (https://www.ncbi.nlm.nih.gov/clinvar/accessed on 24 November 2025) and HGMD (The Human Gene Mutation Database, https://www.hgmd.cf.ac.uk/ac/index.php accessed on 24 November 2025) to identify prior classifications. Multiple lines of evidence are evaluated, including computational predictions, functional studies, segregation analysis, *de novo* status, and phenotype correlation. Based on this evidence, the variant is classified into one of five categories: pathogenic, likely pathogenic, VUS, likely benign, or benign (Table 2). Complex cases should undergo multidisciplinary review involving geneticists, clinicians and genetic counselors. The final classification is reported to the patient, accompanied by genetic counseling. As new data emerge, variants may be reclassified, highlighting the importance of ongoing data management and follow-up.

### 5.1. Variants of Uncertain Significance

The interpretation of VUS and incidental findings in ES presents a significant clinical and ethical challenge. However, these challenges differ, and consequently, the approach to interpretation and decision-making regarding whether to report VUS and incidental findings is also different. A VUS is identified for its potential relevance to the fetus’s phenotype, but the evidence is often insufficient or conflicting, requiring cautious evaluation and ongoing reclassification. In contrast, an incidental finding refers to genetic alterations discovered unintentionally, outside the scope of the primary diagnostic indication, but which may have potential health implications for the fetus or family. Therefore, the term incidental finding does not capture the uncertainty associated with a VUS or inconsistent gene-phenotype correlations in the fetus. The limited phenotypic data available during pregnancy, combined with the urgency of decision-making, complicates both the evaluation and the final reporting of VUS and incidental findings.

Approximately 4–20% of cases in ES yield a VUS, although this proportion is expected to decrease as knowledge advances and variants are reanalyzed [15]. There is broad consensus among international guidelines regarding the reporting of primary findings (including pathogenic or likely pathogenic variants, and VUS in compound heterozygosity with a pathogenic or likely pathogenic variant in recessive genes related to the indication for testing). However, reporting practices for VUS in the prenatal setting vary between laboratories, and there is currently no universal consensus on the optimal approach for their evaluation and disclosure [16]. Decisions regarding the reporting of VUS require careful evaluation of molecular data, evolutionary conservation, gene expression profiles, and in silico predictions. Multidisciplinary review is essential for assessing the potential pathogenicity of VUS, particularly when a strong genotype-phenotype correlation exists [17]. The ACMG guidelines suggest that VUS should not be used in clinical decision making. However, in selected cases, reporting highly suspicious VUS (“hot VUS”) has influenced parental decisions and led to clinically actionable outcomes [18]. The possibility of reclassifying VUS with emerging evidence further highlights the importance of longitudinal data management and follow-up.

### 5.2. Incidental Findings

In contrast, decisions about reporting incidental findings are guided by clinical actionability, ethical considerations, and patient preferences, rather than solely by phenotype correlation. Several international scientific societies have established guidance on the reporting of incidental findings in genomic testing within the prenatal setting. The American College of Medical Genetics and Genomics (ACMG) and the Canadian College of Medical Geneticists (CCMG) both recommend reporting clinically significant variants—classified as pathogenic or likely pathogenic—in genes with high penetrance that are associated with moderate or severe childhood-onset conditions [19,20]. Importantly, neither society supports reporting carrier status, reflecting a focus on actionable findings with clear clinical impact.

In the prenatal setting, genome/exome-wide sequencing presents additional challenges due to the potential implications for both the fetus and the family. The framework proposed by Vears & Amor [21] in 2022 provides structured guidance for reporting incidental in prenatal sequencing, emphasizing informed consent, patient autonomy, and the clinical relevance of findings. This framework advocates a baseline analysis with optional additional layers, balancing the benefits of early detection against risks such as overdiagnosis, anxiety, or unnecessary interventions. Complementing this, the joint position statement from the International Society for Prenatal Diagnosis (ISPD), the Society for Maternal-Fetal Medicine (SMFM), and the Perinatal Quality Foundation (PQF) emphasizes careful evaluation and reporting of genome-wide sequencing results in the fetal setting, highlighting the need for standardized policies, ethical oversight, and interdisciplinary collaboration [22].

Together, these recommendations reflect an emerging international consensus on the responsible implementation of prenatal genomic testing. By focusing on clinically actionable findings and integrating structured frameworks with professional guidelines, prenatal sequencing can provide meaningful information for patient care while respecting ethical principles.

In summary, the focus, interpretation, and decision-making processes differ fundamentally between VUS and incidental findings. Current guidelines emphasize the importance of comprehensive pre- and post-test counseling to inform expectant parents about the possibility and implications of both VUS and incidental findings. In some cases, these findings may reveal actionable conditions or carrier status, potentially influencing reproductive decisions or prompting further family testing. However, the potential for anxiety and uncertainty underscores the need for a cautious and individualized approach to their interpretation and reporting. As genomic technologies continue to evolve, ongoing research and dialogue will be essential to refine reporting practices, optimize patient counseling, and ensure that the full potential of genomic medicine is realized in the prenatal setting.

## 6. Selection Strategies for Gene Interpretation in ES

### 6.1. Main Gene Selection Strategies

Prenatal exome sequencing (ES) is a widely accepted method for determining whether a monogenic disorder underlies specific fetal structural anomalies. After negative results from QF-PCR and CMA, the entire coding region of the genome is typically sequenced. However, data analysis is usually restricted to a subset of genes to enhance diagnostic yield and reduce the number of incidental findings. At one end of the spectrum, all genes can be analyzed; at the other, laboratories establish virtual panels focusing on specific diseases or phenotypes. Between these two extremes, three common approaches are used in prenatal exome interpretation.

The first is the use of morbid OMIM genes, approximately 5000 in number, referred to as the “clinical exome”. OMIM (https://www.omim.org/, accessed on 24 November 2025) is a compendium of human genes and genetic phenotypes, containing information on all known Mendelian disorders with over 26,000 genes, with a focus on the relationship between phenotype and genotype. The OMIM Morbid Map Scorecard includes 7611 phenotypes (updated 30 April 2025) for which the molecular basis is known and 4978 genes with a phenotype-causing mutation [23]. Prenatal phenotypes are not separately noted.

The second is the R21 pathway of PanelApp Genomics England (https://panelapp.genomicsengland.co.uk/panels/478/, accessed on 24 November 2025), which restricts analysis to genes related to severe early-onset diseases for which a prenatal phenotype is apparent. It is a public compendium of genomic panels continuously curated based on external reviewers and England’s Genomic Medicine Service and evaluated by the NHS Genomic Medicine Service [24]. For example, the fetal anomalies panel, called R21 (latest signed version v6.85), employs a targeted panel of 1514/2332 genes associated with severe early-onset human monogenic diseases. It is divided in three lists: the green list is used to interpret variants, while the genes constituting the amber and red lists differ on the level of evidence supporting the association with fetal structural anomalies or suggesting that variation in these genes is benign regarding said phenotype [25].

The third approach involves a personalized panel that follows a phenotype-first strategy, prioritizing the detailed description of specific fetal anomalies to guide the selection of genes to interpret after ES. This is guided by the Human Phenotype Ontology (HPO) terms (https://hpo.jax.org/, accessed on 24 November 2025) and it is referred to as the HPO-driven exome. The HPO project provides an ontology of over 18,000 medically relevant phenotypes, disease-phenotype annotations, and the algorithms that operate on these [26].

Finally, while exome slices (i.e., restricted analyses using virtual panels, morbid gene lists, or phenotype-driven subsets) are limited to predefined gene sets, whole exome analysis allows for the evaluation of all genes. Since OMIM morbid genes are directly applicable to clinical practice, non-included genes are not immediately usable but may enable the discovery of novel disease genes. Exome slices reduce variant burden, improving interpretability and turnaround time while limiting incidental findings. They are useful when fetal phenotypes are well defined but risk missed diagnoses if relevant genes are excluded. Full exome and slice-based approaches are complementary, with choice guided by clinical context and acceptable diagnostic uncertainty.

### 6.2. Gene Strategies Selection Compared

In our center we aimed to assess whether the causative variants found through clinical exome sequencing in fetuses affected by selected structural anomalies would also be detected using either the PanelApp-R21 approach or HPO terms [27]. Over a nine-year period (2016–2024), ES was performed in 206 pregnancies with selected fetal structural anomalies at our center. The whole exome was sequenced, and morbid OMIM genes were interpreted as part of the clinical ES approach. In total, 79 causative variants were found across 78 fetuses, and 19 incidental findings were reported. Primary findings were then reviewed to determine whether they would have been identified using PanelApp-R21 or HPO-driven gene selection. The same comparison was applied to the 19 incidental findings.

The analysis revealed that among the 79 causative genes identified through interpretation of morbid OMIM genes, PanelApp-R21 was able to detect 76 genes, corresponding to 96%, while HPO-driven analysis detected only 56 genes, or 71%. For the 19 incidental variants, PanelApp-R21 identified eight genes (42%), whereas HPO terms identified only one (5.3%). Some phenotypic findings lacked a match within HPO, abnormal subclavian artery morphology, abnormal brain stem, small vermis and corpus callosum, delayed sulcation, abnormal olfactory sulci, anomalous costal vertebrae and body stalk.

In conclusion, retrospective use of HPO terms significantly reduces the detection of incidental findings to 5.3%, but at the cost of missing 29% of primary findings. By contrast, the PanelApp-R21 approach misses only 4% of primary findings while reducing incidental findings by 58%.

## 7. Conclusions

Advances in prenatal molecular diagnostics, particularly CMA and ES, have substantially improved the detection and interpretation of genetic abnormalities before birth. These technologies provide complementary strengths: CMA is highly effective for identifying copy number variants, whereas ES enables in-depth analysis of monogenic disorders. Their clinical indications differ, as CMA has largely replaced prenatal karyotyping in most of its former applications and is strongly recommended in the presence of fetal structural anomalies, while ES is primarily used to investigate suspected monogenic disorders associated with selected fetal anomalies. The clinical utility of both approaches depends not only on technical performance but also on accurate phenotypic assessment, standardized variant classification, and careful reporting to ensure that the results are meaningful and actionable for families. Given the relatively stable diagnostic yield of CMA across anomaly types, a concurrent CMA–ES approach appears justified in selected fetal anomaly groups such as skeletal dysplasia, recurrent anomalies, fetal akinesia, multisystem anomalies and hydrops. Interpretation remains a major challenge, particularly for variants of uncertain significance and incidental findings with unclear or unexpected implications for pregnancy management. Multidisciplinary collaboration involving fetal medicine specialists, geneticists, and genetic counselors is essential to support appropriate test selection, result interpretation, and family-centered counseling, particularly in the context of time-sensitive prenatal decision-making. In the context of ES, agnostic gene selection strategies appear preferable, given the limited and evolving understanding of genotype–phenotype correlations in the prenatal setting. Ongoing advances in bioinformatics, systematic data sharing, gene curation, and interpretative frameworks will further enhance diagnostic yield, reduce uncertainty, and support more precise, informed, and ethically responsible prenatal care.

## Figures and Tables

**Table 1:** Comparison between prenatal CMA and ES.

Feature	Chromosomal Microarray (CMA)	Exome Sequencing (ES)
Type of Alterations Detected	Copy number variations (CNVs), microdeletions, microduplications	Single nucleotide variants (SNVs), small insertions/deletions (indels)
Resolution	Detects large-scale genomic changes (typically > 50 kb)	Detects single base changes and small variants in coding regions
Detects Aneuploidies	Yes	No (unless incidentally detected)
Detects Balanced Rearrangements	No	No
Detects UPD/Homozygosity	Yes (with SNP arrays)	No
Detects Mosaicism	Limited (depends on level and platform)	Limited (depends on variant allele frequency)
Detects Monogenic Disorders	No	Yes
Detects CNVs	Yes	No (unless CNV-calling algorithms are used)
Diagnostic Yield	~6–17% in fetuses with anomalies and normal karyotype	~10–30% in fetuses with anomalies and normal CMA
Turnaround Time	Faster (3–5 days)	Longer (1–3 weeks)
Clinical Use	First-tier test for structural anomalies	Second-tier test when CMA is normal but anomalies persist
Limitations	Misses single-gene disorders, balanced rearrangements	Misses CNVs, non-coding variants, mitochondrial mutations

**Table 2:** Variant classification following ACMG guidelines and clinical interpretation.

Variant Classification	Definition	Clinical Interpretation
Pathogenic	There is strong, consistent evidence—from studies and case data—showing the variant causes disease	Clinical relevance depends on whether the variant explains the observed phenotype. Actionability is context-dependent.
Likely Pathogenic	There is strong but incomplete evidence that the variant causes disease	
Variant of Uncertain Significance (VUS)	Evidence is unclear or conflicting, and more data over time are needed to determine the variant’s significance	Typically not used to guide clinical management unless there is a strong phenotype correlation.
Likely Benign	There is strong evidence indicating it is not associated with disease, though it does not meet the full criteria for benign classification.	Not clinically significant and not used to guide medical management.
Benign	Not to be associated with disease. Typically, common in the general population and lack functional or clinical evidence of pathogenicity.	

## Data Availability

No new data were created or analyzed in this study. Data sharing is not applicable to this article.
